# Nutrition and Vulnerable Groups

**DOI:** 10.3390/nu11051066

**Published:** 2019-05-14

**Authors:** Amanda Devine, Tanya Lawlis

**Affiliations:** 1 School of Medical and Health Sciences, Edith Cowan University, Joondalup, Perth WA 6027, Australia; 2 Discipline Nutrition and Dietetics, University of Canberra, Canberra, ACT 2601, Australia; Tanya.Lawlis@canberra.edu.au

Food insecurity is a complex ‘wicked’ problem that results from a range of unstable and uncertain physical, social, cultural, and economic factors that limit access to nutritious food. Globally, 800 million people are undernourished, around 1.9 billion are overweight/obese, and 2 billion have micronutrient deficiency [1]. This, in part, is explained by changes in food production and manufacturing and their impacts on climate change [2], the retraction in economic climates, increases in food prices, and, in some regions, reduced food availability and access [3,4]. Vulnerable groups include, but are not limited to, migrant populations, Indigenous peoples, elderly populations, pregnant women, those with disabilities, homelessness people, young children, and youth. Poor nutrition during significant periods of growth and development and throughout life impacts long-term health outcomes; increases non-communicable disease prevalence, healthcare costs, and disease burden; and negatively impacts economic and human productivity [5]. This special edition has brought together a variety of articles, some positioned in developing countries where disease burden is high and food insecurity issues impact the growth and development of young children while also negatively affecting adults, specifically their mental and physical health. This issue, Nutrition and Vulnerable Groups, reports novel strategies to address individual, household, and community food security, and draws together quantitative and qualitative research that has attempted to address the challenges of food security while considering the complexity of the problem, the need for locally-driven and scalable solutions, and policy implications. 

The double burden of disease exists in many countries, especially in developing countries and those transitioning to Western-style diets. Factors influencing infant feeding practices in Haitian children have been examined, and despite a high prevalence of malnutrition and poor adherence to the World Health Organization’s recommendations exacerbating malnutrition, factors including low maternal education and greater family size have been negatively associated with infant nutritional status [6]. Households that experience child stunting have simultaneous issues with overweight and obese parents, the odds of which relate to the level of food insecurity and appear to be greater in those with mild food insecurity [7]. This may be explained by marginally greater access to food, but food of poor nutritional quality, explaining the juxtaposition of the disease burden. Other vulnerable food insecure groups, such as refugees, are experiencing additional impacts of increased obesity including metabolic syndrome. An increased likelihood of this condition has been related to older age, synonymous to years of exposure, as well as younger marital age [8]. Author recommendations suggest large-scale community intervention programs to tackle obesity as well as cultural change to increase age at marriage.

In both developing and developed countries, socioeconomic status is a known driver of food insecurity and the association with increased Body Mass Index (BMI) in children and adults is clear. This issue examines children who have experienced abandonment and are being supported by the welfare state, and how sociodemographic factors negatively impact children’s body size and body shape satisfaction [9]. Authors recommend body image awareness as a consideration in obesity prevention programs. Moreover, poor academic performance in low socioeconomic adolescents has been related to greater body size and fatness, alone or in combination with diet and exercise patterns, and seems more likely to occur in males than females [10]. Poor food choice or limited access to nourishing food, such as fruit and vegetables, is associated with food insecure populations [11,12], especially youth, and is explained by a lack of economic means, education, food availability, access, and other socioeconomic factors. Support mechanisms, including programs to increase access to healthy food, are paramount for vulnerable communities, and this issue provides evidence of the importance of food pantries [13], as well as school and university settings [14,15]. However, in some countries popular restaurants that support low income families and provide cheap, energy-dense foods to support the cultural aspects of the traditional food supply simultaneously increase the risk of chronic disease [16].

Similarly to developing countries, cities and neighborhoods in developed countries are experiencing a greater emergence of vulnerable populations, thus requiring an informed workforce to support these communities. This workforce needs to identify modifiable factors that can be incorporated into future schemes and food security interventions in order to efficiently manage food shortages and address drivers in the immediate and broader geographical locations [17,18]. A greater understanding from the workforce is required, as evidence suggests a divergence in views between those who address the problem and those with the lived experience of food insecurity [19]. Therefore, more engagement and attention to those with the lived experience is required to inform interventions. Strategies outlined in this issue to influence nutritional intake include greater access to local food pantries [13], educational interventions for children and adults [20,21], and increased local food production and livestock keeping [22].

Food literacy is among the key components required to improve food security, as evidence in this issue highlights the lack of understanding by food-insecure households about food labelling, product attributes, and food choice [12]. Authors in this issue outline a framework that builds the capacity and capability of the charitable food organization workforce, through the inclusion of the university or higher education sector, to support their training needs in food literacy [15].

To better understand the practice and policy environment of the broader food system, barriers and enablers have been examined [17]. This issue has outlined novel applications of a Systemic Innovation Lab, which capture initiatives within a defined local geographical area that support community food security [18]. This innovative system examines systems change. Initiatives that had a greater number of characteristics to reinforce a better way of working to address food insecurity or had strategies and systems to implement place-based change were identified. Those without these characteristics were identified, and strategies were co-designed by the community to improve the initiative to more comprehensively address food security. Community and government buy-in, relationship building, and education were among the strategies required to improve systems change. 

The diverse articles in this special issue highlight the complexity and extent to which nutrition-related issues may impact vulnerable and marginalized groups. The impact of over- and undernutrition is not specific to one group or area, as similar problems have been identified in developing and developed countries and between rural and urban areas. As seen by the various findings and recommendations, not only is more work in this area required but the translation of this work to practice and policy is imperative if we are to address the issues impacting upon the nutrition and health of those experiencing vulnerability.
